# Outcome Priorities Among Evidence-Based Dementia Caregiving Programs: A Closer Look

**DOI:** 10.1093/geroni/igab046.028

**Published:** 2021-12-17

**Authors:** Sara Powers, Alyssa Ciancibello, Rachel Schaffer, David Bass, Morgan Minyo

**Affiliations:** 1 Benjamin Rose Institute on Aging, Cleveland, Ohio, United States; 2 Cleveland State University, Cleveland, Ohio, United States

## Abstract

Currently, the Best Practice Caregiving website provides information on 231 published studies from 44 dementia caregiving evidence-based programs that have demonstrated beneficial outcomes for dementia caregivers within health care and community-based settings. Across all programs, a total of 34 biopsychosocial outcomes were identified. Supported by the commonly used stress-related frameworks (e.g., Stress-Health Process, Cognitive Behavioral Theory) for which the programs were developed, the most frequently utilized program outcomes included: 1) Caregiver stress, strain, and/or burden (84.1%); 2) Caregiver depressive symptomology (79.5%); and 3) Caregiving efficacy, skills, and/or confidence (63.6%). The least common programmatic outcomes included: 1) Access to support information/Community service use (9.1%); 2) Unmet needs (6.8%); and 3) Respite/break from care (2.3%). The lesser utilized outcomes provide critical insight into current evidence-based programmatic priorities and ways in which professionals can seek to fill gaps in dementia caregiving interventions. Discussion will also focus on future directions of caregiver-related outcome assessments.

